# Neural processing of olfactory-related words in subjects with congenital and acquired olfactory dysfunction

**DOI:** 10.1038/s41598-020-71245-x

**Published:** 2020-09-01

**Authors:** Akshita Joshi, Pengfei Han, Vanda Faria, Maria Larsson, Thomas Hummel

**Affiliations:** 1 Smell and Taste Clinic, Department of Otorhinolaryngology, TU Dresden, Fetscherstrasse 74, 01307 Dresden, Germany; 2 Gösta Ekman Laboratory, Department of Psychology, Stockholm University, Frescati Hagväg, 9A 106 91 Stockholm, Sweden; 3 Faculty of Psychology, Southwest University, Chongqing, China; 4 Department of Psychology, Uppsala University, Uppsala, Sweden; 5 Centre for Pain and the Brain, Department of Anaesthesiology, Perioperative and Pain Medicine, Boston Children’s Hospital, Harvard Medical School, Boston, MA USA

**Keywords:** Olfactory system, Sensory processing

## Abstract

Olfactory loss can be acquired (patients with a history of olfactory experiences), or inborn (patients without olfactory experiences/life-long inability to smell). Inborn olfactory loss, or congenital anosmia (CA), is relatively rare and there is a knowledge gap regarding the compensatory neural mechanisms involved in this condition. The study aimed to investigate the top-down olfactory processing in patients with CA or idiopathic acquired anosmia (IA) in comparison to normosmia controls (NC) during expectancy and reading of odor-associated words. Functional magnetic resonance imaging was used to assess brain activations in 14 patients with CA, 8 patients with IA, and 16 NC healthy participants during an expectancy and reading task. Words with strong olfactory associations (OW) (e.g. “banana”) or with little or no olfactory associations (CW) (e.g. “chair”) were used as stimuli and were presented with a block design Analyses were conducted to explore the brain activation in response to OW expectancy or OW reading between groups (CW as baseline). During the expectancy condition of OW, IA and NC groups showed stronger activation in posterior OFC extending to right insula, caudate region and frontal medial OFC respectively. Whereas during the reading condition of OW, CA patients showed stronger activation in posterior OFC extending to the insula. Increased activation of higher-order brain regions related to multisensory integration among CA patients suggests a compensatory mechanism for processing semantic olfactory cues.

## Introduction

In humans, the causes for compete smell loss (anosmia) are due to either acquired or congenital causes. In contrast to acquired anosmia which the sense of smell is impaired later in the life, congenital anosmia (CA) is a rare condition which is characterized by a life-long lack of olfactory perception and the aplasia or hypoplasia of the olfactory bulb^1^.

Stimulation with either odor molecules or olfactory associated non-chemical cues (e.g. pictures, words, metaphors) can activate the central olfactory system, representing the bottom-up and the top-down pathways for olfactory processing. For bottom-up process, odor molecules bind to olfactory receptors before olfactory signals are transmitted via olfactory bulb and are further processed in multiple olfactory related brain regions (e.g. piriform cortex, amygdala, orbitofrontal cortex, insula, hippocampus, anterior cingulate cortex)^2–4^. On the other hand, during top-down processing, the retrieval of cognitive information related to an odor occurs without the existence of a physical stimulus^5^. These top-down activations involve the olfactory-related as well as higher-order brain regions^2,6–9^.

Patients with olfactory dysfunction demonstrate decreased brain activation in response to odor stimulation, indicating a disrupted bottom-up olfactory process^10,11^. Moreover, several brain imaging studies have suggested alterations of the top-down olfactory process among patients with olfactory loss. For example, Flohr et al.^12^ found that patients with acquired smell loss are unable to vividly image odors with a given odor-associated cue, and exhibited enhanced brain activation in the dorsal lateral prefrontal cortex and the precuneus regions mainly involved in working memory. Using blocks of words with strong olfactory associations, Han et al.^13^ investigated the reading of odor related words among a group of patients with acquired olfactory loss. Specifically, during the word priming, patients had increased activation of the lexical-semantic related areas during expectation of words with olfactory association. Combined, these studies suggested that patients with olfactory loss have changed neural responses in the olfactory cortex during processing of olfactory information. However, research on the neural processing of olfactory information among CA are limited.

To date only a few studies have investigated the structural and functional alterations in CA. While reduced gray matter volume in olfactory related brain regions are found in acquired anosmia, CA is associated with increased gray matter volume in the primary olfactory area and the orbitofrontal cortex^14,15^. One recent study showed that CA exhibited audio-visual multisensory enhancement, which suggested a compensation for complete lack of olfactory input^16^. If and to what extent brain responses during top-down olfactory processes are altered in CA is unknown. The current study aimed to investigate brain processing of odor-related words in CA and compare that to patients with acquired idiopathic anosmia (IA), and normosmic controls (NC). We hypothesized that IA and NC subjects show more activations in olfactory associated areas because of their pre-existing olfactory associated semantic knowledge whereas activations in CA subjects were expected to be significantly lower as compared to others because of their complete lack of olfactory experience^17^.

## Materials and methods

### Participants

Participants were recruited from the resident of Dresden area (control participants) and the Smell and Taste Clinic, Department of Otorhinolaryngology, University Hospital Carl Gustav Carus, Dresden (patients). All participants received the “Sniffin’ Sticks” olfactory test^18^. A composite odor threshold, odor discrimination and odor Identification score (TDI score) was used to classify normal olfaction (TDI > 30.5) and anosmia (TDI < 16.5). In order to ascertain anosmia in CA patients, olfactory event related potentials were recorded, and in none of the patient’s olfactory event related potentials were detected^19^. CA subjects were diagnosed with lack or hypoplasia of olfactory bulb and a life-long olfactory dysfunction without other known etiology. Patients with idiopathic anosmia (IA) were those patients with no cause for their olfactory dysfunction was found after detailed clinical investigations (including medial history questionnaires, psychophysical olfactory testing, olfactory pathways morphology assessment)^20^. In addition, participants completed the German version of Beck Inventory [ranging from normal state (1–10) to extreme depression (over 40) II]^21^ and the Montreal Cognitive assessment (ranging from 0 to 30)^22^ for assessing the level of depression and executive functions, respectively.

A total of 40 participants took part in the study. Of those, eighteen were control participants with normal olfaction (NC, mean age 49.2 years; SD 12.2; 10 females), 14 were patients with congenital anosmia (CA, mean age 37.4 years, SD 18.9; 7 females) and 8 patients with idiopathic anosmia (IA, mean age 56.4 years; SD 10.8; 4 females, disease duration ranging between 9 and 108 months). The study was approved by the Ethics committee of the medical faculty at the Technical University of Dresden. The experiment was conducted according to the Helsinki declaration. All participants provided written informed consent.

### Study design

For our experimental design, 36 words with strong olfactory association (OW) and 36 words with little or no olfactory association i.e. control words (CW) were presented to the participants lying in the scanner. Apart from the 24 new words as displayed in Table 1, for convenience of later analysis some words were randomly repeated to have a block time of 20 s each.

**Table 1:** Words shown to the participants in the scanner (words in German, with English translation in brackets).

Olfactory associated words	Non-olfactory associated control words
Fisch (fish)	Nadel (needle)
Popcorn (popcorn)	Stein (stone)
Zimt (cinnamon)	Schlüssel (key)
Karamel (caramel)	Teller (plate)
Senf (mustard)	Aufzug (elevator)
Leder (leather)	Schloß (lock)
Vanille (vanilla)	Kugel (bullet)
Zigarre (cigar)	Schere (scissors)
Wein (wine)	Brille (glasses)
Käse (cheese)	Halsband (collar)
Rose (rose)	Schachspiel (chess)
Ananas (pineapple)	Stuhl (chair)
Gummi (rubber)	Ventilator (fan)
Knoblauch (garlic)	Bildscirm (screen)
Anis (aniseed)	Spiegel (mirror)
Pfirsich (peach)	Hefter (stapler)
Menthol (menthol)	Wasser (water)
Schokolade (chocolate)	Handy (mobile)
Gras (grass)	Batterie (battery)
Orange (orange)	Eimer (bucket)
Erdbeere (strawberry)	Uhr (clock)
Kaffee (coffee)	Tasche (bag)
Banane (banana)	Tür (door)
Schweiß (sweat)	Glas (glass)

We chose the words with higher olfactory association as reported by Han et al.^13^. Briefly, 50 words with olfactory association and 50 words with little or no olfactory association were screened and rated by experts (PH, TH, JA, IC). Through a pilot study, 18 normosmics were asked to rate the randomly presented OW and CW words for the degree of olfactory association using a numerical scale ranging from 0 to 5. Combining with the ratings from expert selection, CW had a mean score of 0.4 (SD 0.3) whereas OW had a mean rating score of 3.2 [(SD 0.9); t (17) = 13.5, *p* < 0.001]^13^.

**Figure 1:**
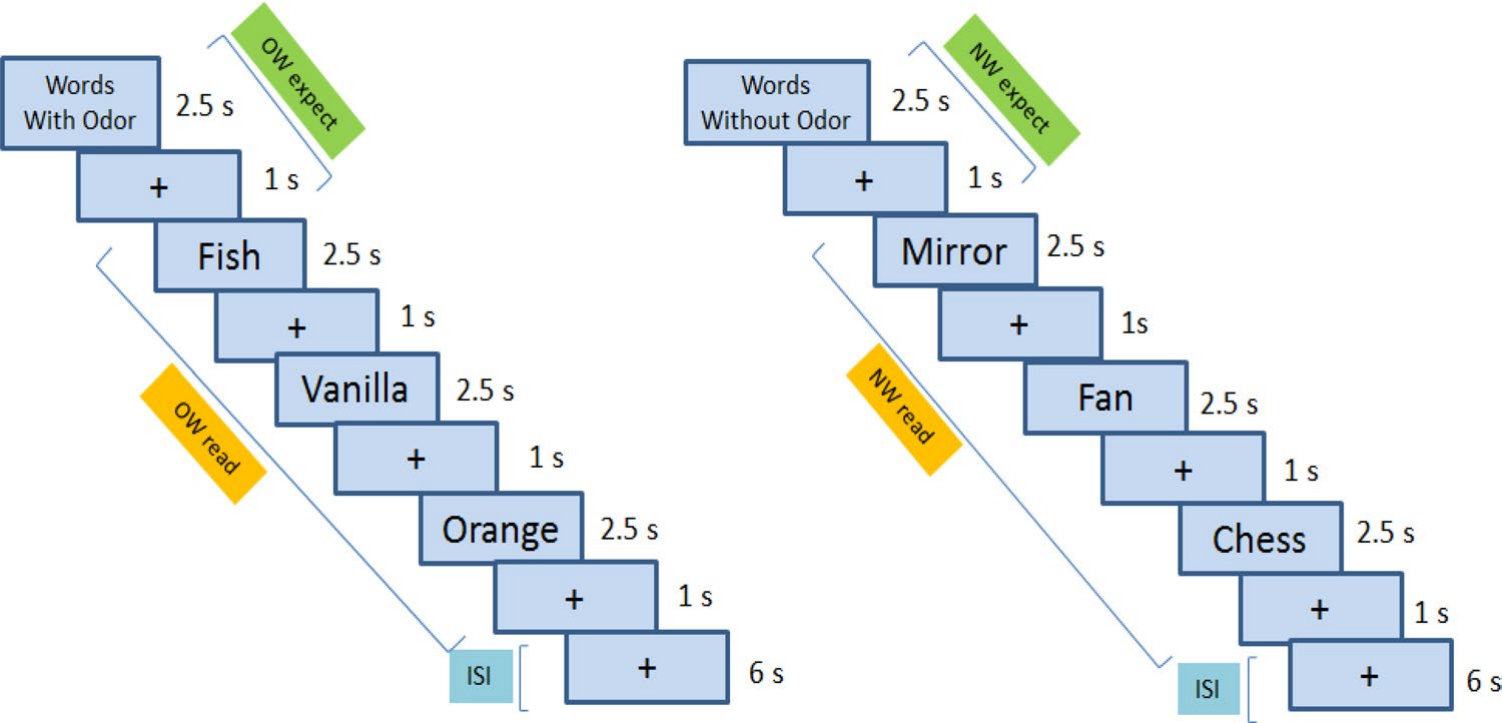
fMRI experimental block design with expectancy task (3.5 s) and reading task (10.5 s); “+” as fixation cross; “ISI” as inter-stimulus interval (6 s) and “s” as seconds.

The participants were instructed to covertly read the instructions and words. Cueing prior to word blocks was adopted to guide participants to (1) focus on the olfactory aspects of the displayed words (2) induce an expectation for the following words; and (3) to clearly separate the OW from the CW blocks. Olfactory related semantic differences were chosen as a criterion to differentiate between conditional activation. However, no control on the word frequency was marked on. The word length (e.g. number of characters in each word) was taken into consideration during selection, however, no statistical analysis was performed on this. Specifically, the experimental run contained 24 blocks in total with 12 blocks each of OW and CW; displayed in an alternating pattern. For each block, the expectation was induced with a slide showing for 2.5 s with the term ‘Words with smell’ (German: ‘Wörter mit Duft’) or “Words with no smell” (German: ‘Wörter ohne Duft’) followed by a 1-s fixation cross, making the expectation task for 3.5 s. The reading phase included three OW or CW presented for 2.5 s each, with 1-s intervals between words, making the reading task for 10.5 s. During inter-block intervals, a fixed cross was shown for 6 s. Each block was of 20 s. The order of the words within each block was randomized among participants. In the complete experiment we had 36 OW + 36 CW words in total scan time of 480 s = 8 min ((12 + 12) × 20 s/block). A simplified diagram of the fMRI design is depicted in Fig. 1.

### Imaging data acquisition and preprocessing

Functional and structural brain images were acquired on a 3-T MRI scanner (Siemens Prisma, Erlangen, Germany) equipped with an 8-channel head coil. A total of 220 functional images were collected per individual using a T2 single-shot echo-planar imaging (EPI) sequence: TR = 2000 ms, TE = 40 ms, 90° flip angle^13^, voxel size 3 × 3 × 3.75 mm, no interslice gap, 192 × 192 mm field of view. A high-resolution structural T1 image was acquired using a 3D magnetization prepared gradient rapid acquisition gradient echo (MPRAGE) sequence (TR = 2530 ms, TE = 2.34 ms, 256 × 256 mm field of view, voxel size 1 × 1 × 1 mm).

SPM12 (statistical parametric mapping) was used to analyze the functional MRI data, which is a MATLAB (The Mathworks Inc., Natick, MA, USA) based software from Welcome Trust Centre for Neuroimaging, London, UK. Default settings were used for pre-processing of data which included—realignment, unwarping, co-registration, segmentation, smoothing and normalization. For all the subjects, head movement artifacts were further removed using ArtRepair software (version 4, Stanford University)^9^, after which neuroimaging data of one control subject was discarded due to excessive movement. In the end, data set included functional images of 14 CA, 8 IA and 16 NC participants.

### fMRI analysis

On the single-subject level, the conditions for OW expect and OW read were calculated as follows: OW_expect_ = (OW − NW)_expect,_ and OW_read_ = (OW − NW)_read_. Further, on the group level the contrast images from each individual were subjected to a random effect analysis to test specific research questions: (1) one-way ANOVA analysis was used to test between-group differences regarding OW expect; (2) the one-way ANOVA analysis was used to test between-group differences regarding OW read. Age, sex, and BDI scores were controlled in the models in the SPM 2nd level model. Significant brain activation was searched on the whole-brain level. To control for multiple statistical testing within the entire brain, we maintained a cluster-level false-positive detection rate at p < 0.05 using an initial voxel-level threshold of p < 0.001 with a cluster extent (k) empirically determined by Monte Carlo simulations (n = 1,000 iterations), by means of AlphaSim procedure^23^. This was done using the REST toolbox (https://www.restfmri.net/forum/REST_V1.7)^24^. A minimum cluster size (number of contiguous voxels) was determined for each specific contrast to achieve a cluster-level Family-Wise Error corrected p < 0.05, and were reported as part of the results. Significant brain regions were labelled and reported with the AAL toolbox^25^ . The activation levels (contrast estimates) in significant clusters were plotted for each group (NC, IA, CA) using the plot function in SPM.

### Statistical analyses for behavioral data

Behavioral and socio-demographic measurements (“Sniffin’ Sticks” test score; BDI score; MCAT score) were analyzed using IBM SPSS version 2.4 (SPSS Inc., USA, Chicago) using one-way ANOVA, including age and sex as co-variables of no interest. The significance level for all the statistical tests was set at p < 0.05 unless specified. Results are represented as means ± standard deviation (SD).

## Results

### Characteristics of participants

The socio-demographical and psychophysical measurements for each group were shown in Table 2. Age of CA was significantly smaller compared to the other two groups. Patients with IA had highest BDI scores compared to NC and CA groups. The sex distribution, taste spray score or the Montreal cognitive assessment test score were not different between groups.

**Table 2:** Socio-demographical and psychophysical information for normal control (NC), congenital anosmia (CA) and the idiopathic anosmia (IA) groups.

	NC (N = 16)	CA (N = 14)	IA (N = 8)	Comparison
Age (years)	49.2 ± 12.2^a^	37.4 ± 18.9^b^	56.4 ± 10.8^a^	p < 0.05
Female/male (n)	10/6	7/7	4/4	n.s
Odor threshold	8.6 ± 2.4 ^a^	1.5 ± 1.5 ^b^	0.4 ± 0.2 ^b^	p < 0.001
Odor discrimination	12.8 ± 2.2 ^a^	5.7 ± 1.7 ^b^	5.6 ± 3.6 ^b^	p < 0.001
Odor identification	14.2 ± 1.7 ^a^	4.8 ± 2.0 ^b^	3.9 ± 2.6 ^b^	p < 0.001
TDI score	35.6 ± 3.8 ^a^	12.0 ± 2.7 ^b^	10.7 ± 6.0 ^b^	p < 0.001
Taste sprays	3.7 ± 0.6	4.0 ± 0.0	4.0 ± 0.0	n.s
MoCA	27.0 ± 2.1	26.6 ± 2.8	25.4 ± 1.7	n.s
BDI	2.5 ± 2.4 ^a^	2.8 ± 2.9 ^a^	7.0 ± 7.2 ^b^	p < 0.05

Comparison p values indicate main effect of ANOVA, superscripts with different letters (a, b) Indicate significant difference in post-hoc comparisons.

*TDI* combined odor threshold, discrimination and identification score, *MoCA* Montreal cognitive assessment test. *BDI* Beck depression inventory test, *n.s.* not significant.

### fMRI results

#### Difference between control and patient groups during expectation of OW

**Figure 2:**
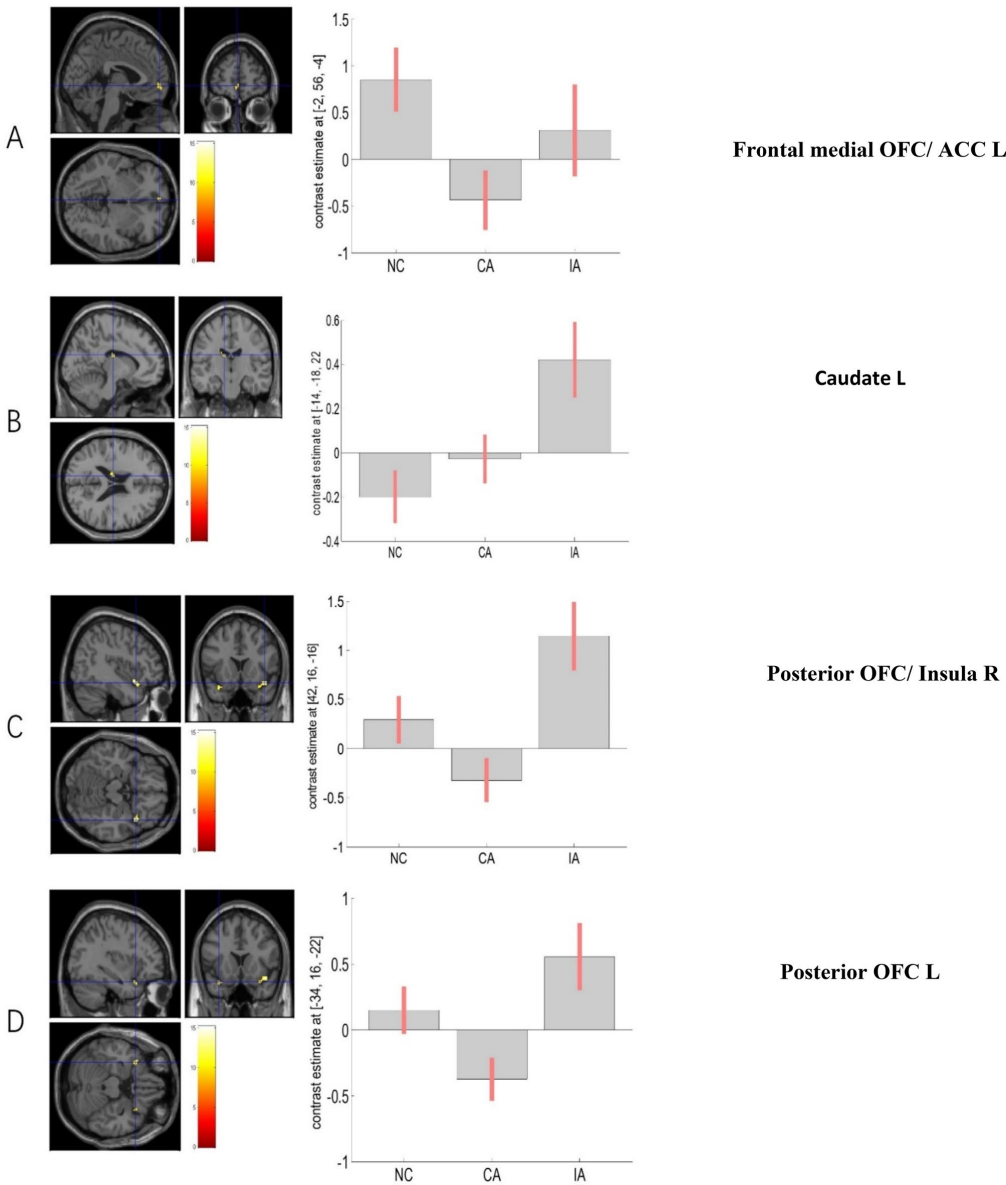
Neural responses showing the main group effect during OW expectation in (**a**) left frontal medial OFC; (**b**) left caudate; (**c**) posterior OFC/right insula; (**d**) left posterior OFC. Brain maps were threshold at p_uncorrected_ ≤ 0.001 in combination of a cluster size determined using the Monte Carlo simulations (n = 1,000 iterations) following the AlphaSim procedure, and visualized on a template (ch2better.nni) provided in SPM12. Bar charts display the contrast estimates for the illustrated regions.

During expectation of OW, significant main effect of group was observed in the right posterior OFC extending to insula, the left posterior OFC, left caudate and anterior cingulate cortex (ACC) (Fig. 2; Table 3). The pairwise between-group comparison showed stronger activation of the frontal medial OFC extending to left ACC among NC compared to CA participants, and stronger activation of the posterior OFC extending to insula among IA compared to CA patients. Besides, the IA patients demonstrated significant stronger activation in the bilateral caudate as compared to NC participants during OW expectation (Table 3).

**Table 3:** Between-group comparisons during expectation of OW.

	k	F/T value	x y z			Region
Main effect	63	15.14	42	16	− 16	Posterior OFC/Insula R
	20	12.65	− 34	16	− 22	Posterior OFC L
	37	11.48	− 14	− 18	22	Caudate L
	27	10.12	− 2	56	− 4	Frontal medial OFC/ ACC L
NC > CA	295	4.50	− 2	56	− 4	Frontal medial OFC/ ACC L
IA > NC	178	4.79	− 14	− 18	22	Caudate L
	40	4.17	22	− 24	24	Caudate R
IA > CA	117	5.43	42	16	− 16	Posterior OFC/Insula R
	46	4.72	− 34	16	− 22	Posterior OFC L

Whole brain F or T maps were thresholded at uncorrected p < 0.001 and cluster size k > 10 voxels; For clusters with multiple peaks only the highest T value is reported; MNI coordinates are presented in x, y, and z. L, left hemisphere; R right hemisphere. Brain regions labelled with AAL toolbox (https://www.gin.cnrs.fr/en/tools/aal-aal2/).

#### Differences in brain activation between control and patient groups during reading of OW

By applying the corrected threshold (p < 0.001 and k > 18 voxels), there was no significant activation of the main effect during reading OW. We further compared NC vs CA, and IA vs CA, separately. With a corrected threshold (p < 0.001 with cluster size > 43 voxels), the CA patients showed significantly stronger activation of the posterior OFC extending to the insular cortex compared to NC participants (peak MNI coordinates 36 16 − 16, T = 4.48, k = 92). There was also stronger activation of the left occipital cortex in CA as compared to IA patients (peak MNI coordinates − 34 − 82 36, T = 3.89, k = 85). No other significant activation was observed from the between-groups comparisons.

## Discussion

The current study investigated neural processing of words with olfactory associations in patients with life-long olfactory loss (congenital anosmia, CA) in comparison with a group of control participants with normal olfaction (NC) and a group of patients with acquired olfactory dysfunction (idiopathic anosmia, IA). Most importantly, the CA group, having never sensed any olfactory stimuli, showed stronger activations in the posterior OFC, extending to insular cortex compared to NC participants during OW reading. The activation in the OFC region is similar to what has been observed in both healthy controls and patients with acquired olfactory loss where reading of olfactory associated words led to similar activations^7,13^. During expectation of OW, the IA patients demonstrated stronger activation in the posterior OFC extending to insula as compared to the CA patients. The OFC is relevant for the processing and integration of information from different sensory modalities^26^. Also, activation in the OFC supports the interaction between the odor related word cues and their respective odor percepts^27^. In comparison to NC, IA patients when expecting the olfactory associated words also showed major activations in caudate, which can be interpreted for its involvement in executive processes^28^ specifically, in goal-directed actions. Moreover, in comparison to CA, NC group showed activations extending to ACC, which indicate its key role in attention^29,30^ and in working memory tasks^31^^.^ NC, IC, and CA seem to use distinct strategies when it comes to anticipating words with olfactory associations.

During OW reading, stronger activation of a very similar cluster in the posterior OFC extending to insula was found among CA as compared to NC groups. As stated above, this region is involved in multisensory integrative processing, that receive information from the olfactory, gustatory and visual sources^32–34^. Unlike the expectation condition where no direct odor-related cues were shown, displaying OW could initiate neural processing of word related semantic and olfactory information more efficiently. Although CA patients have a life-long deprivation of olfactory perception, their knowledge regarding other chemical inputs (e.g. gustatory and trigeminal input) as well as the semantic meaning of the OW remains intact. Besides, CA patients have been shown to exhibit slightly enhanced abilities for non-chemical multisensory (e.g. audio-visual) integration compared to people with intact olfaction^8^, indicating an existing compensatory mechanism. Therefore, stronger activation during OW reading among the CA group may reflect the process of multisensory integration, involving semantic comprehension. The exact process behind the increased activation among CA remains to be explored. Stronger activation was also found in the occipital cortex among CA compared to IA during reading of OW, possibly indicated an enhanced attention paid to the olfactory-related words among CA patients.

A number of fMRI studies have reported similar patterns of brain activation during olfactory memory tasks cued by non-odorous objects such as images or words^6,35,36^ and during the processing of physical olfactory stimuli. Brain activity during representation of sensory stimulus without direct external stimulus (mental imagery) has been studied in various modalities including visual, auditory and tactile. In general, regions associated with mental imagery were found to be those regions associated with perception in the same sensory modality^37,38^. In the present case one would expect participation of primary and secondary olfactory areas, given that these participate in olfactory perception^39,40^. The presently observed activation of OFC when reading the OW, can be related to the odor imagery approach as shown in previous studies. There activation in the right OFC, associating imagery with the perception of physically present odors was related to the experienced realness or “vividness” of an olfactory image^40,41^. In our study, given the visual sources, integration of visual and olfactory information occurs in OFC, where the odor percepts were linked to their respective names. Djordjevic et al.^2^ also reported activation of the insular cortex as a result of odor imagery. Neuroimaging studies suggest that a number of factors could modify activation of these olfactory brain regions. Among these possible effects are: increased respiratory amplitude, due to sniffing^42^, attentional demands^43^, lexico-semantic processing of words^7^, or cross modal associative learning^44^. All in all, olfactory top-down processing has a significant role in encoding or recalling of learned information^45^, which results in anticipation of an odor or processing of odor-associated cues^13^. Therefore, based on the present results it appears that there is overlap of neural processing in terms of both bottom-up and top-down olfactory representation.

There are a few limitations applied to the current study. First, the small sample size. Given the scarcity of CA cases, studies on this group of patients are typically small (i.e. less than 20 patients). Second, the breathing was not monitored during the MRI scan. The possible alteration of breathing in patients^46^ may introduce noise as variable that affect the observed brain responses^10,39^. Thirdly, we did not explore the association of the presented words with foods. Such an association might explain some of the overlapping activations in the 3 groups of patients; fourthly, for reasons of study design, olfactory words and control words were not evaluated for their valence and their association with edibility which also might impact the processing of words.

In conclusion, our results demonstrate different neural responses during expectation and reading of words with strong olfactory associations among people with acquired anosmia, congenital anosmia and normosmia. Increased activation of the higher-order brain regions related to multisensory integration among CA during reading of olfactory related words may suggest a compensatory mechanism for processing of semantic olfactory cues.
